# The Role of Interleukin 6 in Periodontitis and Its Complications

**DOI:** 10.3390/ijms25042146

**Published:** 2024-02-10

**Authors:** Małgorzata Mazurek-Mochol, Tobias Bonsmann, Martyna Mochol, Agata Poniewierska-Baran, Andrzej Pawlik

**Affiliations:** 1 Department of Periodontology, Pomeranian Medical University in Szczecin, Powstancow Wielkopolskich 72, 70-111 Szczecin, Poland; malgorzata.mazurek@poczta.onet.pl (M.M.-M.); martyna.mochol@poczta.onet.pl (M.M.); 2 Institute of Biology, University of Szczecin, Felczaka 3c, 71-412 Szczecin, Poland; agata.poniewierska-baran@usz.edu.pl; 3 Department of Physiology, Pomeranian Medical University in Szczecin, Powstancow Wielkopolskich 72, 70-111 Szczecin, Poland

**Keywords:** periodontitis, interleukin 6, inflammation, gingiva

## Abstract

Interleukin 6 (IL-6) is a pleomorphic pro-inflammatory cytokine that is strongly associated with local as well as systemic inflammatory processes. Its role in physiological and pathogenic processes throughout the human body has been the subject of numerous studies in recent years. Measurements of the IL-6 levels in gingival crevicular fluid (GFC), as well as in serum, can be important diagnostic and prognostic factors in periodontal diseases (PD) and in assessing their impact on a range of related inflammatory diseases. This narrative review explores the significant role of IL-6 in patients with periodontitis and its association with other widespread inflammatory pathologies.

## 1. Introduction

Periodontal disease (PD), also called gum disease or periodontitis, affects all integral parts of the periodontium, from the gingiva and periodontal ligament to the cementum and alveolar bone [1]. Increasing attention is being paid to the oral and dental plaque microbiota and its influence on systemic diseases, as well as general health [2,3]. Periodontitis is a disease initially caused by the accumulation of biofilm and plaque, which leads to bone resorption and loss of the supporting structure of the teeth, and that affects more than half of the adult population worldwide [4]. The initiators of this destructive process are periodontal bacteria, such as *Porphyromonas gingivalis*, *Tannerella forsythia*, *Aggregatibacter actinomycetemcomitans*, etc. Pro-inflammatory cytokines, which are upregulated by the activity of these bacteria, play a key role in destructive processes [5,6]. One of the best known and studied cytokines among them is IL-6, which acts in various physiological and pathological processes ranging from the development to the progression of intra-oral inflammatory processes, including PD.

Interleukin 6 (IL-6) is a lymphocyte chemoattractant factor that can be secreted by a wide variety of cells, such as immune cells, including activated T-cells, B-cells and myeloid immune cells, including macrophages and dendritic cells, as well as relevant non-immune cells, like keratinocytes, endothelial cells and fibroblasts [7,8]. IL-6 belongs to the interleukin-6-type cytokines, together with IL-11, LIF (leukemia inhibitory factor), OSM (oncostatin M), CNTF (ciliary neurotrophic factor), CT-1 (cardiotrophin-1) and CLC (cardiotrophin-like cytokine) [9]. Three new members, IL-27, IL-35 and IL-39, have been recently added to this family [10,11]. Receptor activation, using both membrane and soluble IL-6 receptors, triggers important intracellular signaling pathways and acts via signal transducers, like glycoprotein 130 (gp130), LIF receptor and OSM receptor, leading to the activation of the Janus kinase/signal transducer and activator of transcription (JAK/STAT) cascade, as well as the mitogen-activated protein kinase (MAPK) cascade [12].

IL-6 regulates dendritic cell and B-cell differentiation, but it also has the ability to stimulate T-cell differentiation and therefore plays a key role in regulating the T-cell subsets, e.g., the activation of IL-17-secreting T-helper cells, and the balance between the IL-17-secreting T helper cells and regulatory T-cells, enhancing the immunological response. Importantly, in a non-inflammatory state (in the absence of IL-6), transforming growth factor beta (TGF-beta) activates immunosuppressive and regulatory T-cells. This pro- and anti-inflammatory cytokine stimulates IL-2 receptor expression and secretion and a cascade of other pro-inflammatory interleukins [13,14]. As an inflammation-associated effect, IL-6 also can play important role in the hematopoiesis process and in signaling the acute-phase response proteins, e.g., C-reactive protein (CRP) in hepatocyte cells.

Recent studies have shown the important role of IL-6 in the development of atherosclerosis, as well as many immune-mediated diseases, including periodontitis. The relationship between periodontitis and atherosclerosis has also been demonstrated [15,16,17,18,19,20]. Nibali et al. found that an excessive response to IL-6 (combined with the release of active-phase reactants) can contribute to the development of a chronic inflammatory lesion, resulting in the loss of periodontal ligaments and alveolar bone [21,22]. Interleukin 6 is important due to the fact that it not only induces active-phase responses but also plays a role in the development of specific cellular and humoral immune responses through terminal B-cell differentiation, immunoglobulin secretion and T-cell activation. With respect to these facts, it is additionally a modulator from the acute to the chronic phase of inflammation [23]. Indicating a local destructive process, high levels of IL-6 are accompanied by increased osteoclastic activity in the alveolar bone region and an excess of periodontal-pathogenic bacteria [2,3]. Various scientific studies have examined the IL-6 levels in PD in combination with other diseases and have used them as a diagnostic factor in disease grades [5]. For example, the IL-6 levels are measured to determine the correlation between PD and systemic diseases, such as diabetes [24,25], atrial fibrillation (AF) [6], heart disease [26] and oral cancer [27], as well as other factors such as smoking [28,29,30,31,32] and obesity [33]. Furthermore, factors such as radiotherapy, vitamin C application and tocilizumab application, as well as monoclonal antibodies, might have the ability to elevate or depress IL-6 levels, which are in direct correlation with periodontal diseases.

## 2. IL-6 as a Pro-Inflammatory Marker in Periodontitis

The presence of dental plaque bacteria and their products causes local inflammation, tissue damage and, consequently, the development of periodontal disease. As shown, IL-6 plays a key role in the pathogenesis of PD by inducing osteoclast differentiation and bone resorption and inhibiting bone formation [22,34]. Interestingly, even chewing can induce IL-6 and promote the accumulation of gingival Th17 cells [35].

IL-6 can prompt the formation of osteoclasts from precursors at low concentrations, but when present at a high concentration, it primarily stimulates the activation of mature osteoclasts [34]. In addition to its effect on osteoclasts, studies have demonstrated that IL-6 has a connection to the release and activation of MMPs, which may cause pathological extracellular matrix (ECM) breakdown in PD patients whose IL-6 serum levels are higher than normal [5,22,36,37,38].

**Figure 1:**
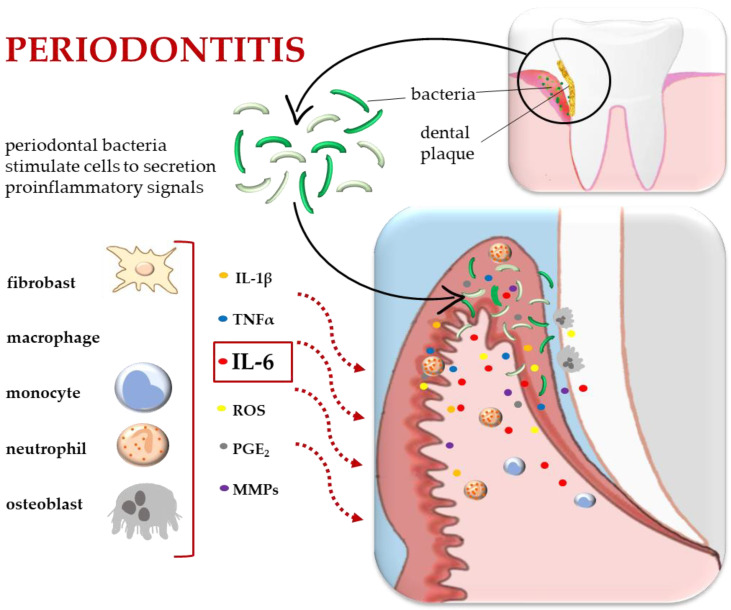
Inflammatory process in periodontitis. The influence of periodontal bacteria on cell activity and cytokine secretion. Abbreviations: IL-1β, Iterleukin 1 beta; TNFα, tumor necrosis factor-α; IL-6, Interleukin 6; ROS, reactive oxygen species; PGE_2_, prostaglandin E2; MMP, matrix metalloproteinases. Illustration adapted some elements from previous work [2].

IL-6 is an important cytokine involved in the regulation of the host response to bacterial infection [39,40,41,42]. Once an inflammatory process is initiated due to a traumatic accident or insufficient hygienic care, the characteristic infiltration of neutrophil cells followed by monocyte cells takes place. After the accumulation of inflammatory cells, various cytokines, such as interleukin-1β (IL-1β), tumor necrosis factor-α (TNF-α) and interferon-γ (IFN-γ), are produced during the inflammatory process, but IL-6 is one of the most prominent interleukins (Figure 1) [17].

Evaluation of the quantitative IL-6 values in patients with and without periodontal diseases clearly illustrates the close connection of this pro-inflammatory cytokine and pathological processes related to the periodontium. Becerik et al. [43] showed that local increases in IL-6 levels, e.g., in the gingival crevicular fluid (GCF) and saliva, were correlated with the presence of gingivitis. Furthermore, the quantitative amounts of IL-6 were significantly elevated in patients with severe or progressive forms of PD compared to its initial stage. Between the initial grades (0–1), the IL-6 levels only increase moderately, but between the more severe grades (2–3), the elevation is more dramatic [44]. These observations were also correlated with histological results.

Proinflammatory cytokines, such as IL-1α, IL-1β, IL-6, IL-8, TNF-α and TNF-β, are associated with osteoclastogenesis, a key process leading to clinical periodontitis outcomes [45] and periodontitis-associated inflammatory diseases [46]. The resorption of the alveolar bone and an increase in osteoclastic processes are initiated by IL-6-mediated factors [22,34], which accompany other clinical markers such as increased bleeding on probing (BOP), clinical attachment loss (CAL) and probing pocket depth (PPD). Interestingly, the IL-6 levels decline rapidly in patients after PD treatment, e.g., after non-surgical treatment, patients with different severity grades, along with reduced IL-6 levels, had better clinical parameters, including BOP, CAL and PPD. Zhou et al. [26] presented that after 3 months of non-surgical periodontitis therapy based on oral hygiene instructions (teaching brushing and flossing), as well as scaling and root debridement, all cytokine levels were highly reduced, including those of IL-6. This suggests a direct connection between the role of IL-6 in the progression and recession of periodontitis. Rudick et al. stated that inflammatory cytokine levels, including those of IL-6, can help determine the subclinical health of a patient, showing the transition from health to gingivitis, or periodontitis, prior to clinical presentation [36]. Taking these acknowledgements into consideration, a new standard of periodontal prevention can be formalized, keeping in mind the current lower diagnostic costs. Tissue destruction can be prevented or minimized. Since IL-6 shows a significant increase during active inflammatory processes and rapidly diminishes after successful treatment, IL-6 has a high diagnostic value [36].

## 3. Changes in IL-6 Levels in Relation to Different Co-Factors

Since the pro-inflammatory role of IL-6 in the pathophysiological course of PD is well known and proven, several attempts to downregulate its quantity have been made in recent years. As already mentioned, the gold standard for improving clinical and laboratory inflammatory markers is non-surgical periodontal treatment. In addition to the improvement in oral hygiene and the removal of debris, calculus and pathogenic bacteria amounts manually, different additional methods have been published worldwide. Toraman et al. [25] evaluated the effects of local application of vitamin C, affecting the tissue levels of several inflammatory markers, including IL-6, and the periodontal attachment loss in rats with induced periodontitis. They proved that vitamin C reduces oxidative stress and IL-6 levels, which also leads to a regression in bone resorption. Immunostaining and the serum IL-6 levels showed the positive effect on the periodontal tissue of rats being treated with vitamin C. Chen et al. evaluated the effectiveness of xipayi mouth rinse combined with minocycline on localized aggressive periodontitis and their therapeutic effect on the levels of inflammatory markers, including IL-6 [47]. With the help of this trial, the evidence has proven that minocycline as an antibiotic agent is effective in downregulating pro-inflammatory cytokines, including IL-6. Furthermore, xipayi mouth rinse and its effect on reducing oxidative stress levels also plays a role in the positive outcome of the study.

A crucial factor in the inflammatory processes in the oral cavity is smoking [28,29,30,31]. In many ways, smoking negatively affects oral health and has a negative impact on inflammatory changes. Surprisingly, Thuller et al. depicted that smoking, in contrast to other inflammatory markers such as IL-17 and IL-1β, does not affect the quantity of IL-6 [32]. Apolinário et al. [22] recently investigated the impact of tocilizumab (TCZ), a known rheumatoid arthritis (RA) drug which inhibits IL-6-mediated pro-inflammatory activity, using an antibody with specificity for soluble and membrane-expressed IL-6R. Tocilizumab is a recombinant humanized monoclonal antibody (mAb) shown to inhibit IL-6 signaling by binding to the IL-6 receptor [42]. TCZ in rats with lab-induced periodontitis clearly depicted reduced the bone loss levels and inflammatory signs due to the reduction in IL-6 levels. This acknowledgement strengthens the point that IL-6 plays a vital role in systemic inflammatory diseases in the whole body.

## 4. Relation between IL-6 in Periodontitis and Other Systemic Diseases

**Figure 2:**
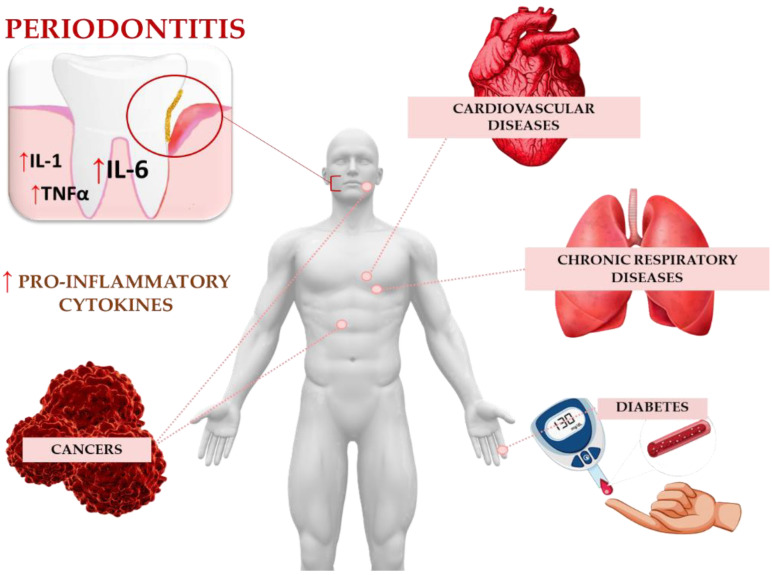
Periodontitis’s connection to systemic diseases. Local inflammation occurs due to pro-inflammatory immune mediators, such as IL-6, involved in disease pathogenesis, such as in diabetes, cardiovascular diseases, chronic respiratory diseases, as well as cancer formation and progression. Illustration adapted some medical elements from Freepik, (www.freepik.com, accessed on 17 January 2024) and Depositphotos EU Limited.

Inflammatory cytokines such as IL-6 not only indicate the grade of PD but are also elevated in many different systemic inflammatory diseases of the human body (Figure 2) [48]. Several proinflammatory immune mediators, including IL-6, are expressed and systemically released into circulation when local inflammation occurs, having an impact on organ systems located elsewhere [49]. Taking this fact into consideration, elevated IL-6 levels in periodontal disease patients are a possible co-factor accelerating the occurrence of inflammation at other distant sites. Considering patients with immune suppression, the importance of lowering their IL-6 levels as a result of treatment is becoming increasingly important [49]. Machado et al. were able to prove this inter-inflammatory connection throughout the human body when examining the serum IL-6 levels in organ-transplanted patients with and without periodontitis [49].

### 4.1. IL-6 in Periodontitis and Diabetes

PD is well known to be in a close pathophysiological relationship with diabetes mellitus (DM) type 2, and IL-6 is indeed a significant proinflammatory cytokine in both DM and periodontitis pathogenesis [50]. Periodontal destruction and diabetes have a synergistic effect on the elevation of inflammatory cytokine levels [50]. Along with bone atrophy, a deeper periodontal probing depth and a higher clinical attachment loss, a steep rise in IL-6 levels has been depicted. These results suggest that diabetes promotes bone loss and tissue destruction by increasing the cytokine levels in the human body [51]. Conversely, well-treated diabetes patients show lower IL-6 levels and a less severe grade of periodontitis. Diabetes and periodontitis are both mediated by complex interactions between the microbiome, inflammation, oxidative stress, genetics, the host immune response and other factors [52]. Despite the extensive knowledge tapped by modern science, the molecular mechanisms of their interdependence and the pathways between diabetes and physiological changes in the periodontal tissue are still unclear. Endocrinologists and diabetologists need to be aware of periodontitis and its impact on glycemia levels and the potential risk of insulin resistance [53]. It follows that patients with periodontitis should also be observed in terms of their risk of developing diabetes [54].

### 4.2. IL-6 in Periodontitis and Cardiovascular Disease

Recent studies have shown the important role of the inflammatory process in the development of arteriosclerosis [15,16,17]. The relationship between periodontal disease and the development of atherosclerosis has also been demonstrated. The link between periodontal disease and atherosclerosis may be related to the effect of bacteria on the blood vessels, where they can initiate inflammation involving pro-inflammatory cytokines, including IL-6 [16,18,19]. The development of inflammation in the vessel walls leads to the initiation of the atherosclerotic process and can cause plaque instability, leading to acute coronary syndromes. IL-6 has been shown to play an important role in the development of the atherosclerotic process, causing increased inflammation in the vasculature, leading to endothelial dysfunction and atherosclerotic plaque instability [19,20]. The atherosclerotic process leads to the development of cardiovascular diseases, especially ischemic heart disease. Another well-known health problem affecting patients in PD is atrial fibrillation (AF), which causes heart failure and stroke. The pathophysiological mechanisms of AF indicate a link with oral health, including periodontitis [6]. Plachokova et al. explored the oral microbiome in periodontitis in relation to disease severity and systemic inflammation [41]. Their data suggest that severe periodontitis may be driven by the oral microbiome and support an inflammatory mechanism behind the association between periodontitis and cardiovascular disease. They showed that the “red complex” and “cluster B” abundances in the periodontal pockets were strongly dependent on the IL-6 levels and the white blood cell count (inflammatory markers) [41]. Zhou et al. studied 75 subjects with both chronic periodontitis (CP) and stable coronary heart disease (CHD) [26]. During the study, they controlled the serum levels of tumor necrosis factor-α (TNF-α), IL-6 and C-reactive protein (CRP), but also the lipid profile markers and white blood cell count. After non-surgical periodontal treatment, all the clinical parameters improved, and the serum levels of IL-6 (as well as TNF-α and CRP) were reduced significantly, which could help to reduce the inflammatory burden of stable CHD subjects.

### 4.3. IL-6 in Periodontitis and Cancer

Interleukin-6 (IL-6) is a crucial component of the cancer microenvironment, and a close relationship has been suggested to exist between cancer and PD [27,55]. The results of a multivariate analysis presented by Kajihara et al. showed that the peripheral blood (PB) concentrations of IL-6 were significantly higher in cancer patients than in the non-cancer patient group and higher in cancer patients with periodontitis than in cancer patients without periodontitis [55]. These results indicated that the presence of periodontitis and cancer synergistically increased the number of Treg cells, and an increased level of IL-6 could be associated with cancer progression. Chronic periodontitis has been especially associated with an increased risk of oral [56] and squamous cell carcinomas [57]. Higher concentrations of IL-6 in the serum and other biological fluids are reported in patients with head and neck squamous cell carcinoma (HNSCC) [58,59]. Importantly, in HNSCC patients at the T3/T4 stages, the number of positive nodal metastases and the advanced-stage IL-6 levels were, respectively, higher [60]. Riedel et al. also confirmed these results, reporting that HNSCC patients had higher serum IL-6 concentrations than healthy controls [61]. In contrast, Andersson et al. did not prove a statistically significant difference in the serum IL-6 concentrations between HNSCC patients and healthy individuals [62]. Either way, these results do not diminish the importance of IL-6 during cancer progression and metastasis. Unfortunately, in PD patients, the correlation between the inflammatory factor levels and the carcinogenesis process is influenced by certain factors, such as tobacco and alcohol use, as well as oral human papillomavirus (HPV) infection, which often limit these results. Duffy et al. decided to check the possible relationship between health-related behaviors and the IL-6 levels in HNSCC patients [58]. The results showed that smoking, weak sleep scores, maturity and advanced tumor stage were significantly associated with higher serum IL-6 concentrations. Interestingly, tumor location, alcohol consumption, BMI and patients’ physical activity, as well as socio-economic indicators, such as education and financial situation, did not have a significant correlation with serum IL-6 concentrations [58]. Elevated serum IL-6 concentrations were also reported in oral squamous cell carcinoma (OSCC), a topographic subtype of HNSCC. As reported by Sato et al., IL-6 is upregulated in the saliva during the treatment of patients with OSCC relative to the level in control subjects [63], and a higher T-stage and clinical stage, deeper bone invasion and larger tumor depth, as well as a lower OSCC patient survival, are correlated with higher IL-6 levels [64].

### 4.4. IL-6 in Periodontitis and Chronic Respiratory Diseases

As was written by Cardoso et al. [48], the oral cavity is a reservoir for pulmonary infection, and there is some evidence that chronic periodontitis may be associated with the risk of pneumonia or chronic obstructive pulmonary disease (COPD) [65,66,67,68]. Theoretically, the oral bacteria that create dental plaque are also present in the saliva and then travel to the lower respiratory tract and lungs, causing an infection. Then, the cytokines and enzymes induced in the inflamed tissues also reach the lungs, where they can stimulate local inflammatory processes [66]. Interestingly, in COPD and chronic periodontitis, the same pro-inflammatory mediators are secreted and accelerate the progression of both pathologies [67,69]. There are also data from a 2-year pilot study indicating that periodontal therapy (in COPD patients with chronic periodontitis) may improve lung function and decrease the frequency of COPD exacerbation [70]. These studies supported an association between chronic periodontitis and chronic respiratory diseases, although large-scale prospective epidemiological studies are still needed.

## 5. IL-6 Gene Polymorphisms in Periodontitis

The regulation of IL-6 production is often linked to genetic factors, including the presence of certain genetic syndromes such as neurofibromatosis type 1 (NF1). Patients with these syndromes are reported to exhibit upregulated IL6 and a higher risk of periodontitis [71]. In addition, there are many known polymorphisms in the promoter region of the IL-6 gene, like -597G/A (rs1800797), -572C/G (rs1800796) and -174G/C (rs1800795) [72,73]. These three single nucleotide polymorphisms (SNPs) in the IL-6 promoter result in individual variations in IL-6 transcription and expression [74]. The -174G/C and -572C/G IL-6 gene polymorphisms increase IL-6 expression, so they may be associated with susceptibility to periodontitis.

### 5.1. Interleukin-6-174G/C Polymorphism in Periodontitis

The associations between this type of polymorphism and PD have been confirmed in studies and meta-analyses [75,76,77,78,79,80]. In 2009, Shao et al. presented the relationship between the IL-6 174 G/C polymorphism and periodontitis as initial meta-analysis results and proved that the GG genotype is related to a high risk of periodontitis, which was confirmed by numerous other reports. In nine studies that included 574 controls and 1093 patients with PD, they discovered that IL-6-174G/C polymorphism is not linked to a risk of chronic periodontal disease, but this allele may raise the risk of aggressive periodontitis [81]. Zhu et al., in a meta-analysis covering 21 case–control studies, proposed that Brazilian and Caucasian populations are more likely to be at risk of chronic periodontal disease if they have the genotype IL-6-174G/G (also known as rs1800795) [82]. In 2010, Costa et al. [77] also observed in a Brazilian population that the IL-6-174G/C gene polymorphism may play a role in chronic periodontal disease. According to Zhao et al. [83], data from a meta-analysis suggest that the IL6-174 G/C polymorphism may be negatively associated with the risk of both general and overall periodontitis. A 2010 study showed that the IL-6-174 G/G genotype was associated with periodontitis in non-smokers and older subjects (>45 years old) in an Indian population [84]. In turn, Stefani et al. showed that a high expression of IL-6 was not associated with the methylation status or -174G/C polymorphism in PD patients [85]. There are also some studies indicating no association between the IL-6-174G/C polymorphism and PD in Caucasian or Asian populations [85,86]. As can be seen, the data vary depending on the origin of the study group and its clinical characteristics.

### 5.2. Interleukin-6-572G/G Polymorphism in Periodontitis

Studies have indicated an association between the IL-6-572GG genotype and periodontal disease, e.g., chronic periodontal disease in Caucasian [87], Chinese [83] and Japanese populations [88], as well as aggressive periodontitis [21]. In 2004, Holla et al. suggested that the -572 G/C polymorphism in the IL-6 gene may be a protective factor associated with the lower susceptibility of Caucasian patients to chronic periodontitis [87]. In turn, the research by Xiao et al. indicates that the frequency of the C allele of IL-6-572 in Caucasians was much lower than that in the Chinese population [74]. There are also data showing that the IL-6-572G/C polymorphism did not show significant associations with the prevalence of periodontitis or periodontal parameters [80], in close agreement with results in a Northwestern Chinese Han population [79]. The IL-6-572GG genotype was linked to chronic periodontal disease and aggressive periodontitis in Shao et al.’s meta-analysis [81]. The inconclusive findings from the aforementioned studies may be explained by variations in the pathogenesis of the two types of PD, as well as genetic predisposition to chronic and aggressive PD.

### 5.3. Interleukin-6-597G/A Polymorphism in Periodontitis

This type of IL-6 polymorphism is probably the least studied. The data available are sparse, but they show some interesting observations. In the Sharma study, they found a statistically significant association between the IL-6-597 gene polymorphism and a chronic periodontitis with type 2 diabetes mellitus (CHPDM) group [89]. The genotype IL-6−597 GA/AA (and allele A) was significantly higher in the controls than in the CHPDM group, so the IL-6−597 gene polymorphism may be protective. This result was in contrast to that of a previous study conducted on a Chinese population, where no significant difference was observed. Interestingly, in a Caucasian population, the IL-6-597 G/A promoter polymorphism showed associations with type 2 diabetes. In 2019, Majumder et al. did not find a statistically significant association of the IL6-597 G/A (rs1800797) gene polymorphism with an increased susceptibility to chronic inflammation of the periodontium in an Indian population [90]. In the European population, the allele frequencies at the -597 G/A polymorphism were also similar in both groups [87]. These data may confirm the hypothesis of a genetic factor being involved in the pathogenesis of chronic periodontitis (CHP) and the idea that IL-6 gene polymorphisms in the regulatory regions may change the expression of this cytokine.

## 6. IL-6 Values in Patients with PD: Treatment Approaches and Disease Prevention

Cytokines, like interleukins (ILs), play a vital role in regulating inflammation, which should eliminate pathogenic bacteria and stimulate the regeneration of affected tissues. However, impaired immunoregulation leads to a prolonged inflammatory response, granulation tissue formation and extensive alveolar bone resorption [22]. Inflammatory conditions, including PD, can regress and return to a physiological state only when the cytokine levels (such as that of IL-6) return to normal [36]. In modern dentistry, the diagnostic tests to help determine the IL-6 levels in gingival crevice fluid are inexpensive and easy to perform, and sample collection is simple. Proper evaluation and knowledge of their value can contribute to a highly effective and preventive way of reducing pathological processes in the periodontium. Furthermore, in addition to improving oral health, a patient’s systemic health can be bettered and/or further disease progression prevented. Tissue destruction, which is highly severe in this context due to the irreversibility of periodontal pathology, can be prevented in accordance with in-office diagnosis. In the case of elevated cytokine levels, prophylactic treatment can be started before the first clinical symptoms appear. In patients who are already affected, IL-6 levels can be considered as an indicator. Several experimental studies have measured the IL-6 values in serum as well as in GCF to prove the effectiveness of periodontitis therapy. In addition to clinical control parameters, diagnostic IL-6 values help clinicians to understand the pathophysiology of a patient’s individual disease, and the probability of regression can be determined with great accuracy [91,92,93]. When clinical parameters are improved and the levels of cytokines are also reduced, the probability of an effective cure is very high. With this premise in mind, attempting to maintain the levels of cytokines, including IL-6, using non-invasive and easy-to-implement methods, such as non-surgical treatment of periodontitis, at a constant physiological level seems to be a strategy with a potential wide-ranging impact on the oral health of patients.

## 7. Conclusions

Inflammation is a natural response of the immune system (IS), caused by many factors, like IS cell–pathogen contact, damaged cells and toxic substances. The oral and dental plaque microbiota and how it affects general health and systemic diseases are receiving more and more attention. The oral mucosa limits the colonization of microorganisms according to the high rotation and secretion of cytokines and anti-microbial proteins, despite the fact that there is strong exposure to many different microbes. Inflammation may develop in some cases as a result of the imbalance between pathogenic and normal oral flora.

Interleukin 6, as a pro-inflammatory cytokine, presents a very broad field for research. It is associated with periodontitis alone, as well as in PD combination with certain human systemic diseases, such as diabetes, cardiovascular disease, chronic respiratory diseases and even cancer. These factors, including IL-6, may trigger acute and/or chronic inflammatory responses, ultimately leading to tissue damage or disease. As it is becoming increasingly clear that IL-6 mediates and accelerates PD, its levels must be tightly regulated. Clinical trials that have attempted to lower IL-6 levels have positively correlated them with the treatment outcomes. Classical non-surgical periodontal therapy based on oral hygiene instructions (teaching brushing and flossing), as well as scaling and root debridement, significantly lowered IL-6 levels, which was associated with positive clinical outcomes, such as an improved CAL, PD and BOP, and inhibited alveolar degeneration. IL-6 plays—as the current science reveals—a key role in PD, and single nucleotide IL-6 polymorphism mutations can aid in determining an increased risk of developing periodontitis.

Severe periodontitis also imposes a large social, economic and healthcare burden, which increases global medical expenses and has a major influence on the human public health system [94]. Knowing the role of IL-6 and its importance in oral health, periodontists can use its diagnostic value in the prevention of periodontal pathology. Since IL-6-174 GG genotypes and the G allele in particular seem to be associated with aggressive periodontitis, and the IL-6−597 gene polymorphism can be a protective type, a detailed diagnosis of patients is important for appropriate treatment planning, as well as for understanding patients’ genetic predispositions.

Pro-inflammatory cytokines activate inflammatory cells and trigger characteristic signaling pathways (NF-κB, MAPK and JAK-STAT). Inflammatory cytokines, such as IL-1β, TNF-α and IL-6 molecules, can potentially serve as biomarkers for many diseases’ diagnosis and prognosis and anti-cytokine therapies. Periodontal diseases are complex systems that cannot be predicted through the investigation of single elements, just like the immune response to periodontal pathogens. The host response (in vivo) activates signaling pathways according to multiple non-microbe or microbe-associated molecular patterns, which means that the immunological response does not result only from individual cytokines or pathways. It is important to note that cytokine responses are only one element in the regulation of the host inflammatory responses. Great hope is placed in mathematical and bioinformatics tools or systems, which can potentially facilitate our understanding of these complex systems and predict the effects of single changes to them [39].

Further studies need to be conducted to thoroughly understand the role of IL-6 in the pathogenesis of periodontitis, both chronic and aggressive. For this reason, it is important to encourage further studies on the interrelationship between inflammation, the oral mucosa and systemic chronic diseases, and more research is still needed on how the combination of these diverse signals is involved in the regulation of immunity at the oral mucosal barrier.

## Data Availability

Not applicable.
